# Exploring the Neural Mechanisms of Acupuncture Stimulation in Autism Spectrum Disorder: Protocol for a Randomized, Sham-Controlled, Multimodal Neuroimaging Trial

**DOI:** 10.2196/97910

**Published:** 2026-10-15

**Authors:** Jaehee Sim, Koung-soon Choi, Dong Hyuk Lee

**Affiliations:** 1 College of Korean Medicine, Sangji University, Wonju-si, Gangwon-do, Republic of Korea; 2 Department of Speech Language Pathology, College of Health Sciences, Sangji University, Wonju-si, Gangwon-do, Republic of Korea; 3 Department of Anatomy, College of Korean Medicine, Sangji University, Sangjidae-gil 83, Wonju-si, Gangwon-do, 26339, Republic of Korea, 82 0337300664; 4 Research institute of Korean medicine, Sangji University, Wonju-si, Gangwon-do, Republic of Korea

**Keywords:** autism spectrum disorder, acupuncture, neuroimaging, EEG, electroencephalogram, fNIRS, functional near-infrared spectroscopy, brain connectivity, multimodal imaging, neural mechanism, randomized controlled trial

## Abstract

**Background:**

Autism spectrum disorder (ASD) is a neurodevelopmental disorder characterized by restricted and repetitive behaviors and disturbances in social communication and interaction. Currently, there is no fundamental treatment for ASD, with only a few medications available for use on a limited basis to manage concomitant neuropsychiatric symptoms. The primary therapeutic goal of such treatments is to alleviate symptoms while maximizing the functional independence and quality of life of affected individuals. Many complementary and alternative medicine therapies have been reported to have effects on ASD symptoms; however, evidence is insufficient to confirm their clinical effectiveness. Acupuncture has traditionally been used as a complementary intervention to manage various neurological and neuropsychiatric diseases, including ASD, in East Asia. However, how acupuncture stimulation modulates neural activity at the macroscale level in individuals with ASD remains largely unknown.

**Objective:**

This study aims to elucidate disease-related brain alterations and explore the acute neural mechanisms underlying acupuncture stimulation in patients with ASD and in typically developing (TD) individuals, using a multimodal neuroimaging approach.

**Methods:**

This study was designed as a randomized, sham-controlled trial. In this stratified 2×2 design, 70 participants, including individuals with ASD (n=35) and TD controls (n=35), will each be randomly assigned to either the verum or sham acupuncture group, resulting in 4 groups. The verum acupuncture group will receive stimulation at specific acupoints clinically indicated for ASD, eliciting a deqi sensation. The sham acupuncture group will receive superficial needling at nonacupoints unrelated to ASD symptoms. Each group will undergo clinical assessments and multimodal neuroimaging measurements, including functional near-infrared spectroscopy and electroencephalography, before and after acupuncture stimulation. The primary outcome will be differences in resting state network metrics and relative band power in frontal areas by disease status. Secondary outcomes will include differences in these neural markers following acupuncture stimulation between the verum and sham groups, reflecting the group × intervention interaction. Exploratory outcomes will focus on identifying neural markers that correlate with clinical indicators of ASD.

**Results:**

Recruitment began in April 2025. To date, 23 individuals with ASD and 21 TD individuals have participated in this study. The recruitment process is expected to continue until December 2026.

**Conclusions:**

ASD presents a significant socioeconomic burden, with no definitive treatment established to date. Although acupuncture is a widely applied intervention for various neurological conditions, including ASD, its large-scale neural mechanisms remain poorly understood. By using multimodal neuroimaging, the present study aims to investigate the immediate neural substrates underlying acupuncture stimulation in patients with ASD. These findings are expected to provide a robust neurobiological foundation for future longitudinal research and the development of evidence-based integrative strategies.

## Introduction

Autism spectrum disorder (ASD) is a neurodevelopmental disorder characterized by deficits in social communication and interaction, in addition to restricted and repetitive patterns of behaviors, interests, and activities [1]. Genetic predispositions and environmental factors during early development play vital roles in ASD etiology [2]. Although clinical features may be detectable in early childhood, diagnosis is often delayed until the later stages of development. According to estimates from the Centers for Disease Control and Prevention’s Autism and Developmental Disabilities Monitoring Network in the United States, approximately 1 in 44 children is diagnosed with ASD [3]. As this disorder manifests early in life and often persists throughout life, it imposes a substantial socioeconomic burden [4,5].

Owing to the lack of sufficient evidence to clarify the underlying mechanisms, no definitive treatment for ASD has yet been established. In conventional medicine, various pharmacological agents have been used; however, these medications primarily target specific symptoms or comorbidities, rather than the core deficits of ASD [6]. For example, although risperidone and aripiprazole may alleviate agitation and irritability in patients, they are commonly associated with adverse effects, including weight gain, nausea, sedation, and hyperglycemia [7]. In addition to pharmacological approaches, behavioral and educational interventions have also been used. Most high-quality behavioral and educational interventions require a substantial time commitment of 20‐40 hours per week and often take a significant amount of time to achieve therapeutic benefits [8,9]. Furthermore, while the use of behavioral interventions, such as applied behavior analysis [10], Treatment and Education of Autistic and Related Communication-Handicapped Children [11], and music therapy, has been steadily increasing, significant unmet needs remain regarding improvements in functional independence and quality of life for individuals with ASD.

In the field of complementary and alternative medicine, between 52% and 74% of patients with ASD have attempted complementary and alternative medicine therapies, with some individuals receiving as many as 7 types of intervention [12,13]. Although these therapies aid in the management of ASD to some extent, there is a lack of sufficient evidence to confirm their clinical effectiveness [14]. Acupuncture is a unique therapeutic intervention in East Asian medicine, widely used to treat ASD. Experimental studies have reported that acupuncture can alleviate ASD symptoms through actions on various biological pathways, including modulation of the gut-brain axis, neurotransmitters, and neurotrophic factors [15-17]. Regarding clinical evidence in humans, 1 study demonstrated that manual acupuncture involving the application of a combination of acupoints led to a significant improvement in verbal communication problems in 97% of patients with ASD [18]. Another study reported that electroacupuncture stimulation significantly enhanced language comprehension and self-care abilities in patients [19]. Despite these clinical advancements, acupuncture research for ASD still faces several limitations, including methodological factors affecting the quality of the study, such as the validity of sham controls, the heterogeneity of ASD across its diverse phenotypes, and the ongoing paucity of research into its underlying neural mechanisms. One of these gaps between clinical outcomes and mechanistic understanding stems from a lack of objective, neural biomarkers capable of reflecting the immediate or longitudinal response of acupuncture on the disease. Therefore, bridging this clinical-mechanistic divide requires direct study of large-scale brain networks, which can be achieved through neuroimaging techniques.

A wide array of neuroimaging modalities, including magnetic resonance imaging (MRI), functional MRI (fMRI), and positron emission tomography, has been extensively used to identify neurobiological markers for the diagnosis and evaluation of therapeutic changes in ASD [20,21]. While fMRI is noninvasive and offers superior spatial resolution relative to other functional neuroimaging modalities, it faces several inherent constraints, including high operational costs, lack of mobility due to bulky instrumentation, and high sensitivity to head-motion artifacts.

Functional near-infrared spectroscopy (fNIRS) has recently emerged as a promising alternative for functional neuroimaging. fNIRS is an optical technique that monitors hemodynamic responses by measuring changes in the concentrations of oxygenated hemoglobin, deoxygenated hemoglobin, and total hemoglobin within the brain tissue, achieved by irradiating the scalp with near-infrared light [22]. fNIRS offers several distinct advantages compared to conventional fMRI; specifically, it provides a relatively higher temporal resolution, is cost-effective, and is remarkably portable. These features together make it highly applicable to “difficult-to-scan” populations, including agitated patients, individuals with claustrophobia, and those with metallic implants, such as pacemakers [23]. Furthermore, its portability and tolerance for head motion are particularly beneficial for pediatric individuals or individuals with cognitive impairment who may struggle to remain still during MRI sessions.

In addition to its well-known temporal advantages, an electroencephalogram (EEG) is a useful tool because it provides direct electrophysiological signals of neural activity with millisecond precision. EEG measurements are based on recording the electric potential differences between electrodes placed on the scalp. These potential differences arise from the volume conduction of ionic currents induced by synchronized postsynaptic potentials across the cell membranes of pyramidal neurons [24]. This allows for a more direct and detailed investigation of rapid cortical dynamics and functional connectivity without the hemodynamic delay or sensory stress associated with the MRI environment.

Both fNIRS and EEG have been used to uncover the neural underpinnings of ASD. For example, fNIRS studies have consistently reported atypical cortical activation or altered connectivity in the prefrontal and temporal regions during resting-state or social tasks in individuals with ASD [25-27]. Similarly, EEG research has identified characteristic spectral power alterations and reduced functional connectivity, particularly in the alpha and gamma bands, which serve as potential electrophysiological biomarkers for this disorder [28-30].

Furthermore, the integrated application of fNIRS and EEG could offer a more comprehensive perspective on brain function than either modality individually. While EEG captures the rapid, millisecond-scale electrical dynamics of neural firing, fNIRS provides localized information on the associated hemodynamic demands by leveraging its enhanced spatial resolution. Integrating these electrophysiological and hemodynamic signals would allow researchers to investigate neurovascular coupling and cross-modal synchronization together. This multimodal approach would be particularly advantageous for ASD research, as it can compensate for the limited spatial resolution of EEG and the hemodynamic delay of fNIRS, ultimately affording an in-depth understanding of the neural mechanisms underlying the disease.

In this context, the present study is designed to investigate disease-related brain alterations by recruiting both typically developing (TD) and ASD groups, using fNIRS and EEG as complementary multimodal neuroimaging techniques. Our main hypothesis is that the ASD group will exhibit altered brain network patterns centered in the frontal regions compared with the TD group, and that these frontal network patterns will change differently depending on the type of intervention. Next, by examining the correlations between these neuroimaging metrics and clinical symptom indices, we focus on identifying neural biomarkers associated with ASD. Beyond establishing this neurobiological foundation, the findings of this study have potential translational value; by mapping individual neural responsiveness, this research could contribute to the development of personalized acupuncture protocols, allowing clinicians to tailor acupoint selection and stimulation parameters based on an individual’s brain network.

Herein, we present a protocol for this randomized, sham-controlled, and parallel-group clinical trial aiming to explore the neural mechanisms of acupuncture stimulation with respect to disease status by comparing TD individuals to those with ASD.

## Methods

### Overview of Study Design

This will be a single-center, block-randomized, sham-controlled, and parallel-group clinical trial conducted at the College of Korean Medicine at Sangji University. This protocol will comply with the Recommendations for Interventional Trials (SPIRIT, Standard Protocol Items: Recommendations for Interventional Trials, Checklist 1) standard Protocol guidelines [31]. This study will investigate the neural mechanisms of acupuncture stimulation in ASD using a stratified 2×2 design, in which participants with ASD and TD individuals are each randomly assigned to either verum (study group) or sham acupuncture (control group), resulting in 4 groups.

After each group is confirmed to participate in the study through screening (visit 0), both fNIRS and EEG scanning, as well as acupuncture manipulation, will be conducted at visit 1. The Korean Childhood Autism Rating Scale 2 (K-CARS 2) will be administered during screening, both as a screening tool and as a measure of symptom severity for ASD. At visit 1, each participant will be assessed twice using fNIRS and EEG (first and second scan) before and after acupuncture stimulation. Finally, clinical indicators of ASD, including social and language skills, will be assessed at visit 1 using the Social Responsiveness Scale 2 (SRS 2) and the Receptive and Expressive Vocabulary Test (REVT). The period between visits 0 and 1 will not exceed 1 week. Participant recruitment was initiated on April 21, 2025, and the study is scheduled to continue until December 2026. A flowchart of the study process is illustrated in Figure 1, while the overall procedure is presented in Table 1.

**Figure 1.**
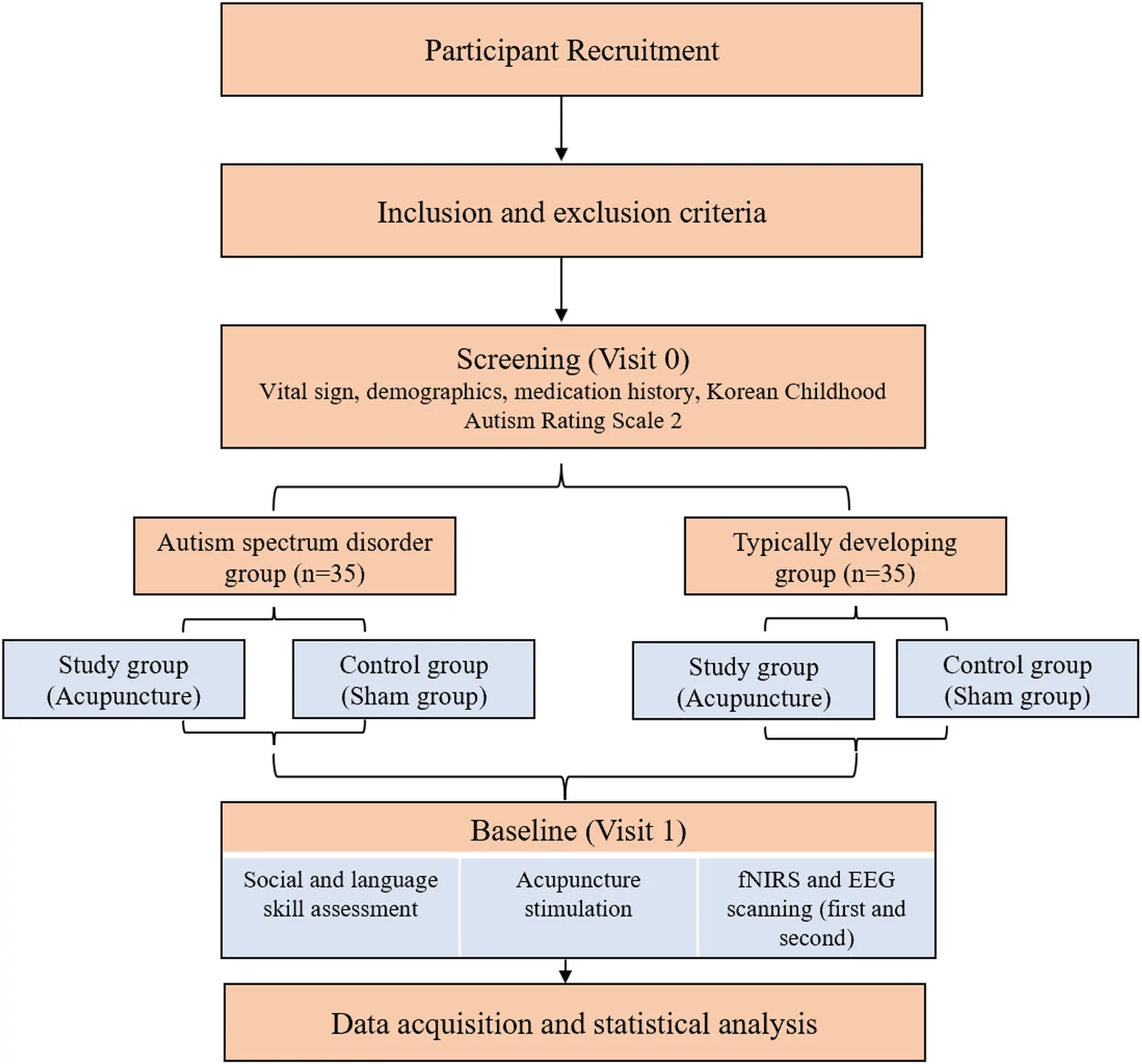
Participant flow diagram for a stratified 2×2 randomized, sham-controlled trial comparing verum and sham acupuncture in children with autism spectrum disorder and typically developing controls. EEG: electroencephalogram; fNIRS: functional near-infrared spectroscopy.

**Table 1.** Overall study procedures by study period in a stratified 2×2 randomized, sham-controlled trial comparing verum and sham acupuncture in children with autism spectrum disorder and typically developing controls.

Measure	Study period	
	Visit 0 (screening)	Visit 1
Enrollment		
Eligibility screen	✓	
Informed consent	✓	
Randomization and allocation	✓	
Intervention		
Verum acupuncture group		✓
Sham acupuncture group		✓
Assessment		
Demographics	✓	
Medical history	✓	✓
K-CARS 2^a^	✓	
SRS 2^b^		✓
REVT^c^		✓
fNIRS^d^ and EEG^e^ scan		✓
Safety		
Vital sign	✓	✓
Adverse events		✓

a K-CARS 2: Korean Childhood Autism Rating Scale 2.

b SRS 2: Social Responsiveness Scale 2.

c REVT: Receptive and Expressive Vocabulary Test.

d fNIRS: functional near-infrared spectroscopy.

e EEG: electroencephalogram.

### Participants and Recruitment Strategy

#### Eligibility Criteria

The inclusion criteria for the TD group are as follows: (1) individuals aged 6 years or older with no neurodevelopmental disorders, matched to the ASD group by age and sex; (2) individuals with no difficulties in verbal communication (eg, reading and writing), who provide written informed consent after receiving a full explanation of the study; for minors, written informed consent must be obtained from a legal representative after a sufficient explanation; and (3) males and females without any neurological or structural conditions that could impair neurodevelopment or cognitive function.

The inclusion criteria for the ASD group are as follows: (1) children or adults aged 6 years or older who have been diagnosed with ASD by a child/adolescent psychiatrist or a pediatrician based on the *Diagnostic and Statistical Manual of Mental Disorders-Fifth Edition* criteria; (2) individuals (or their legal representatives) who have received a full explanation of the study and have voluntarily agreed to participate by providing written informed consent; (3) participants whose symptoms cannot be better explained by other medical conditions or external factors; and (4) individuals deemed capable of successfully completing the study procedures.

The exclusion criteria for the TD group are as follows: (1) individuals with severe neuropsychiatric disorders; (2) a history of neurological or cerebrovascular diseases; (3) the presence of serious systemic medical conditions, such as hepatic, cardiac, or renal diseases; (4) a history of epilepsy or alcohol or drug abuse, or current use of psychotropic medications; (5) the presence of genetic disorders associated with developmental disabilities; and (6) individuals deemed unsuitable for the study at the discretion of the investigator.

The exclusion criteria for the ASD group were as follows: (1) individuals with cerebrovascular diseases such as cerebral hemorrhage or cerebral infarction; (2) individuals with developmental delay or severe cognitive impairment attributable to other primary medical conditions; (3) individuals with serious systemic medical conditions, such as hepatic, cardiac, or renal diseases; (4) a history of epilepsy, alcohol, or drug abuse; (5) individuals currently taking psychotropic medications for comorbid conditions who have had medication changes within the past month; and (6) individuals deemed unsuitable for the study at the discretion of the investigator.

#### Recruitment

Recruitment will be conducted through announcements on university websites and official posts. Participants will be recruited via the on-campus Integrated Support Center for Individuals with Developmental Disabilities and the Department of Korean Neuropsychiatry at the affiliated Korean Medicine Hospital. Additionally, recruitment will be conducted in collaboration with local communities, such as parenting forums and daytime activity support centers for individuals with developmental disabilities in the region. As the participants include minors, written informed consent will be obtained from their legal representatives. In the present study, although most participants are expected to be school-aged children and adolescents (elementary to high school level), adults with ASD may also be included; therefore, no strict age limitations are imposed. Given the male-to-female prevalence ratio of approximately 4:1 in patients with ASD, most participants are expected to be male. However, female participants will also be recruited whenever possible; therefore, sex is not a restricted criterion for inclusion.

#### Randomization and Blinding

A stratified blocked randomization with a block size of 4 will be used to assign participants in both the TD and ASD groups to the intervention or control group in a 1:1 ratio. Randomization will be conducted by an assigned researcher not involved in the assessment or intervention. Participants will be blinded to the type of intervention they receive. Throughout the study, the practitioner will not provide any clues to participants regarding group allocation. Blinding will be maintained throughout the study period. Following the second scan, participants (or their legal guardians) will be asked which intervention they believed they had received (verum or sham acupuncture) in order to assess the adequacy of participant blinding. Unblinding will be permissible only in exceptional circumstances in which knowing the participant’s allocated intervention is essential for their clinical management or safety. Specifically, unblinding may occur if a participant experiences a serious adverse event suspected of being associated with the study intervention. The decision to unblind will be made by the principal investigator after a thorough assessment of the situation. The date, time, and reason for unblinding, as well as the personnel involved, will be documented in detail in the participant’s case report form and reported to the institutional review board (IRB) within 24 hours.

#### Sample Size

For sample size estimation, a power calculation was performed using the G*Power software (version 3.1.9.4; Heinrich-Heine-Universität Düsseldorf) based on a 2-tailed *t* test. With an assumed effect size of 0.8, a significance level (α) of .05, and a power of 0.8, a minimum of 26 participants per group was required. Considering an anticipated dropout rate of 20%, a final sample size of 35 participants per group was deemed appropriate to ensure sufficient statistical power for both groups. The above power calculation was based on the primary comparison—namely, the difference in resting-state network metrics and relative powers in the frontal areas between the ASD and TD groups—for which the planned sample of 35 participants per group provides at least 80% power. As a sensitivity analysis, we also estimated the sample size required to detect the group × intervention interaction (analysis of covariance) within this stratified 2×2 design under a range of assumptions. Assuming a medium effect size (*f*=0.29), the required total sample size ranged from approximately 96 participants (pre-post correlation *r*=0.5) to 67 participants (*r*=0.7). This indicates that the interaction analysis is adequately powered if the pre-post correlation is high, or can otherwise be treated as exploratory given the current sample.

### Study Procedures, Interventions, and Outcome Assessment

#### Study Procedures

All participants will undergo assessments of vital signs (blood pressure, pulse rate, and temperature), demographic information, medical history (past, present, and family history), and current medication use. The K-CARS 2 will be administered for ASD screening at visit 0. Eligible participants meeting all inclusion and exclusion criteria will be scheduled for visit 1.

At visit 1, the investigator will monitor participants’ vital signs and any changes in their medication. The participants will undergo fNIRS and EEG scans twice, before and after acupuncture stimulation. Additionally, clinical indicators of ASD (SRS 2 and REVT) will be assessed to evaluate clinical status. Based on stratified randomization, participants will be assigned to 1 of 2 acupuncture stimulation groups. All acupuncture procedures will be performed by a licensed Korean Medicine doctor with over 3 years of clinical experience. Resting-state fNIRS will be recorded for 5 minutes, and resting-state EEG for 5 minutes, under eyes-closed and eyes-open conditions.

#### Assessment Measures for ASD Severity and Symptoms

The investigator will administer the K-CARS 2 as an ASD screening tool to identify individuals with ASD and effectively distinguish them from those with other developmental disorders, while also measuring the severity of autistic symptoms [32]. Generally, a total score of 30 or higher (out of a maximum of 60) is used as the cut-off for ASD.

Additionally, clinical indicators of ASD symptoms will be evaluated using the SRS 2 and REVT. The SRS 2 is a 65-item parent-report questionnaire designed to quantitatively measure the continuum of typical and atypical social behaviors that covary with ASD symptom severity [33,34]. Each item is rated on a 4-point Likert scale that provides a quantitative measure of social impairment. The REVT will be used to assess language development, a key prognostic factor in ASD, focusing specifically on vocabulary through picture-based tasks [35]. The REVT assesses both receptive and expressive vocabulary abilities, providing standardized scores and percentiles to evaluate lexical development, and identifies potential language delays based on age-specific norms. To ensure objectivity and minimize bias, all clinical assessments will be conducted by an investigator blinded to the participants’ group assignments.

#### fNIRS and EEG Imaging

In the present study, NIRSIT (OBELAB) and SynAmps RT (Compumedics/NFEC-2024-11-300922) will be used for fNIRS and EEG data acquisition, respectively. Resting-state fNIRS data will be acquired for 5 minutes with eyes open, while resting-state EEG will be recorded under 2 conditions: 5 minutes with eyes closed and 5 minutes with eyes open. During the eyes-open recordings, participants will be asked to maintain a relaxed posture while gazing at a monitor. Both fNIRS and EEG data will be acquired before and after the intervention (Figure 2).

**Figure 2.**
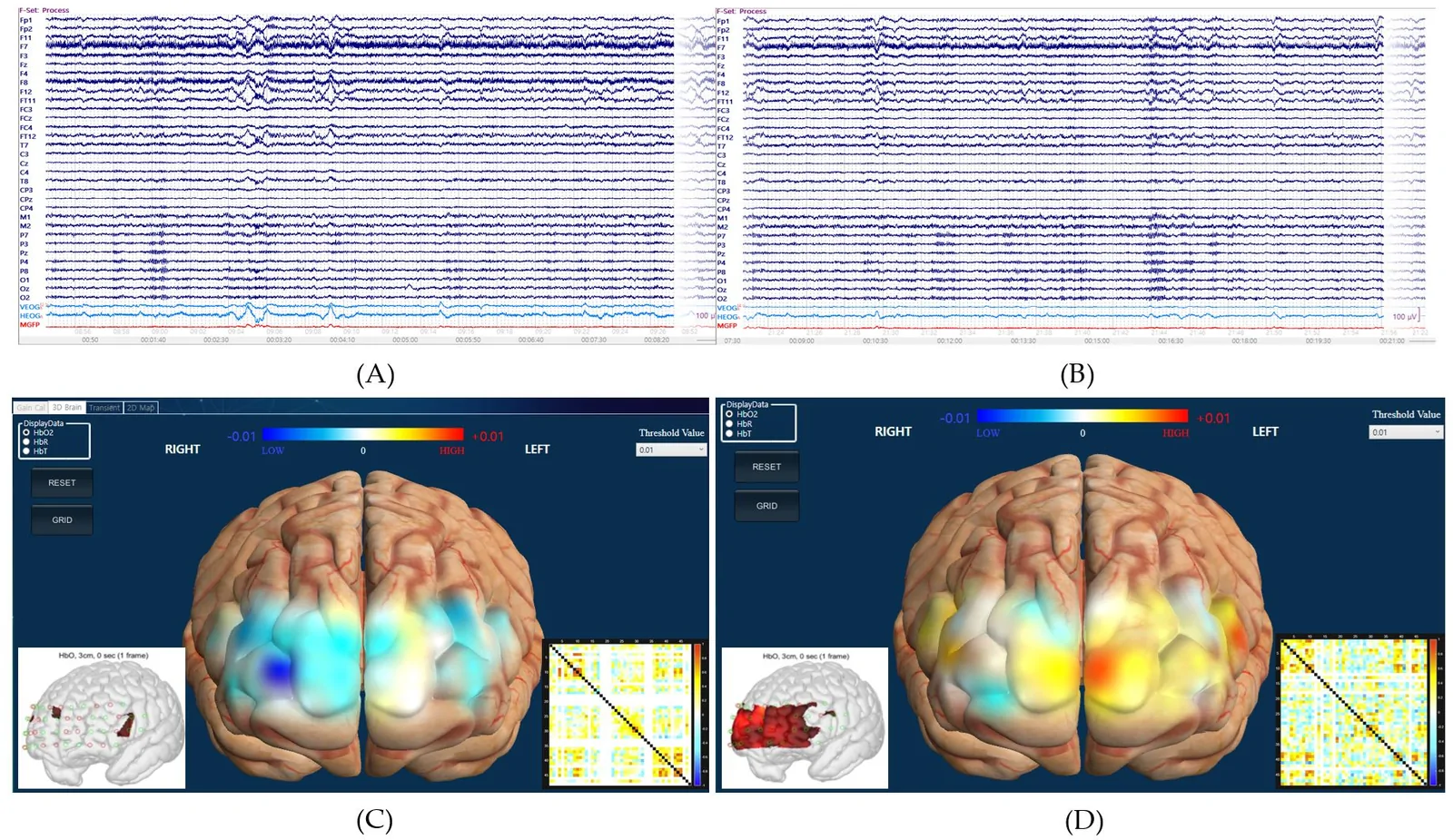
Resting-state EEG and fNIRS scan and connectivity matrix before and after the acupuncture stimulation. (A) EEG scan before acupuncture, (B) EEG scan after acupuncture, (C) fNIRS scan and connectivity matrix before acupuncture, and (D) fNIRS scan and connectivity matrix after acupuncture. EEG: electroencephalogram; fNIRS: functional near-infrared spectroscopy.

The fNIRS equipment comprises a portable neuroimaging modality designed to monitor cerebral oxygenation by delivering near-infrared laser light to the cortex. This system operates using 2 wavelengths, 780 nm and 850 nm, which are safe for human use and allow for the noninvasive measurement of hemodynamic changes. The NIRSIT system features 48 channels primarily focused on the frontal cortical regions, enabling the quantification of regional variations in oxygenated, deoxygenated, and total hemoglobin [36]. Accordingly, the analytical frameworks commonly used in fMRI research, such as graph theory and general linear model analyses, can be effectively implemented in fNIRS. To construct brain connectivity, the 48 frontal channels will be grouped into 8 regions of interest (ROIs) based on Brodmann areas, and an 8×8 connectivity matrix will be computed using Pearson correlation coefficients between ROIs (Figure 3).

**Figure 3.**
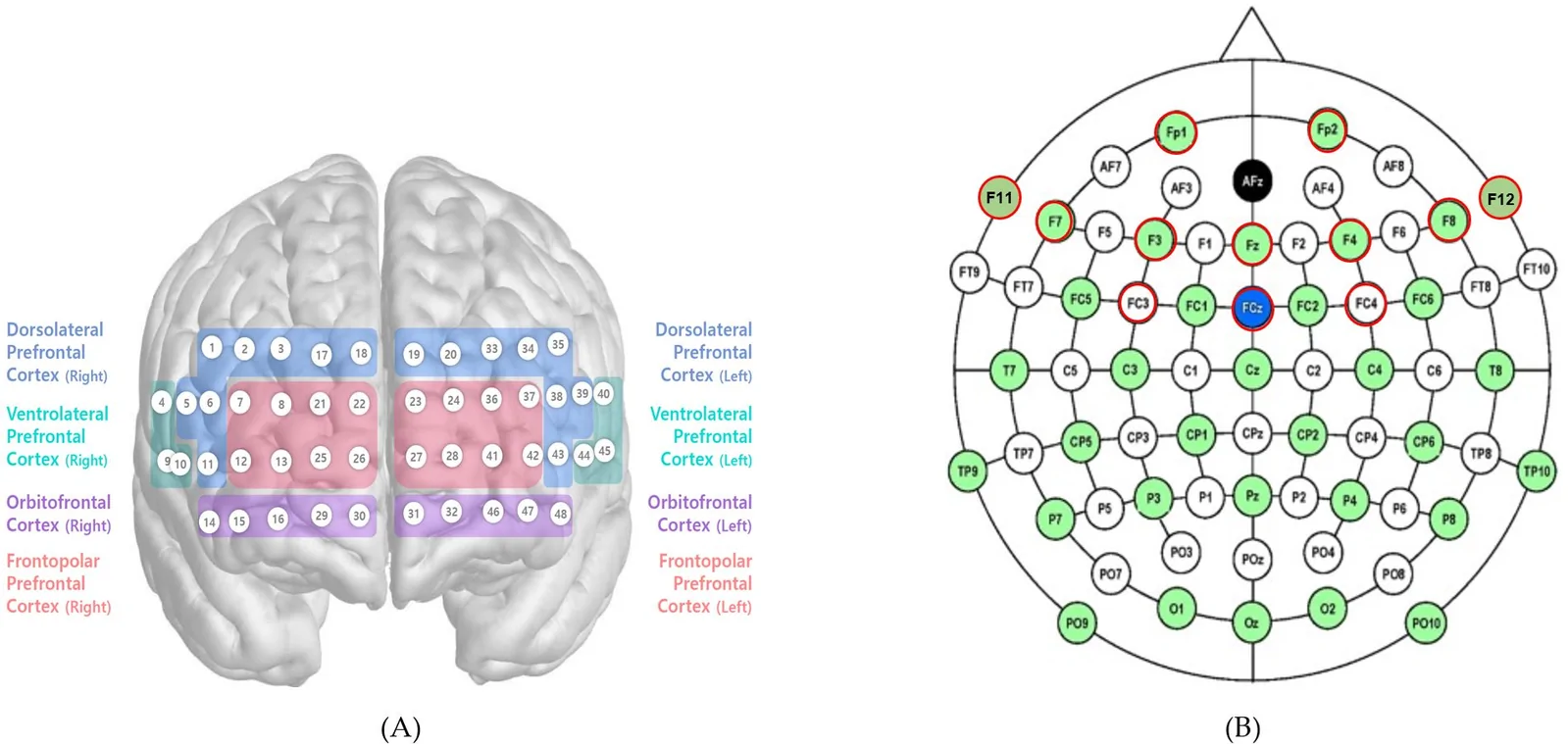
Frontal regions of interest (ROIs) for fNIRS and EEG analyses in this study. (A) Eight Brodmann area ROIs derived from 48 fNIRS channels. (B) Twelve frontal EEG channels (red circles). EEG: electroencephalogram; fNIRS: functional near-infrared spectroscopy.

EEG is an electroencephalography system used to record electrical signals in the brain by detecting neuronal voltage fluctuations. EEG measurements are based on electrical potential differences between electrodes on the scalp, which result from the propagation of current flow induced by synchronized postsynaptic potentials within neuronal membranes. The EEG system uses 32 channels to provide comprehensive coverage of the whole brain. In this study, a 12×12 coherence matrix will be constructed from 12 frontal channels (Fp1, Fp2, Fz, F3, F4, F7, F8, FCz, FC3, FC4, F11, and F12) selected from the 32 channels as ROIs (Figure 3).

Preprocessing and analysis of fNIRS data will be conducted using the NIRSIT Quest tool (v.1.3.1, OBELAB) and custom MATLAB code, whereas EEG data will be preprocessed and analyzed using Curry 9 (Compumedics Neuroscan) software together with the EEGLAB (v2026.0.0; Swartz Center for Computational Neuroscience, University of California San Diego) toolbox in MATLAB. The preprocessing procedures for fNIRS contain channel rejection, rejection padding, conversion to optical density, motion artifact correction, bandpass filtering, and conversion to hemoglobin concentrations [37]. The preprocessing of EEG includes data filtering, bad channel rejection, epoching, baseline correction, artifact removal using independent component analysis, interpolation, and rereferencing [38]. From the resulting connectivity matrices, network parameters, including local efficiency and clustering coefficient, will be computed using the Brain Connectivity Toolbox in MATLAB 2024b (MathWorks).

For each EEG channel, the power spectral density will be estimated using the Welch method (Hanning window, 50% overlap). Relative power will then be computed for the alpha (8‐13 Hz) and gamma (30‐45 Hz) bands as the ratio of the band power to the total power, and used for further analysis. By integrating these 2 modalities, comprehensive information regarding functional neural activity can be obtained from both hemodynamic responses and electrical oscillations of the brain.

#### Interventions

In the study group, verum acupuncture stimulation will be conducted for 5 minutes using stainless steel acupuncture needles (0.20×30 mm, Dongbang Acupuncture) at specific acupoints previously shown to be associated with ASD symptom improvement, including Baihui (GV20), Yintang (EX-HN3), Sanyinjiao (SP6), Zusanli (ST36), Hegu (LI4), and Shenmen (HT7) [39]. To elicit a subtle deqi sensation, a mild manipulation of the needle will be applied, consisting of gentle lifting-thrusting (to a depth of 1‐2 cm) and twisting-rotation (90°‐180°), an approach considered appropriate and tolerable for pediatric individuals. After stimulation, a Deqi Sensation Questionnaire will be administered. For the control group, sham acupuncture will be performed using the same stainless-steel needles at nonacupoints unrelated to ASD symptoms, located within 1 cm of the corresponding verum acupoints on the head, face, and limbs. This will involve superficial needling with an insertion depth of only 1‐2 mm per point, followed by needle retention for 5 minutes. In this sham condition, the needles will remain static, and no attempt will be made to induce the deqi sensation.

Superficial needling is classified as a form of minimal acupuncture [40]. In the present study, the characteristics of the acupoints, depth and intensity of stimulation, and deqi sensation were considered the primary determinants of acupuncture efficacy. Therefore, minimal acupuncture, which lacks these features, was used as the sham control. However, this needling may still activate cutaneous mechanoreceptors, potentially eliciting minor nonspecific neural responses. Thus, the sham condition in this study should be considered an active control rather than an inert placebo. This form of sham-acupuncture has previously been established and applied in various clinical studies [41-44]. Furthermore, as this study involved minors with ASD, relatively small needles were selected to enhance participant compliance and ensure comfort throughout the research process.

#### Outcome Measures

This study aims to characterize disease-related differences in brain networks between the 2 groups, to elucidate the acute neural mechanisms of the acupuncture response, and to identify the specific neural substrates associated with the clinical characteristics of ASD. The primary outcome of this study will be defined as differences in resting-state frontal brain network parameters and relative powers of alpha and gamma bands in the frontal region according to disease status (ASD vs typical development). Secondary outcomes include differences in these neural markers between the 2 groups before and after acupuncture stimulation (verum vs sham acupuncture), reflecting the group × intervention interaction within this stratified 2×2 design. Exploratory outcomes will focus on identifying brain connectivity or network parameters associated with clinical indicators of ASD, such as the K-CARS 2, SRS 2, and REVT scores.

### Data Collection, Management, and Analysis

#### Data Collection and Adverse Event Monitoring

Demographic information (age, sex, height, and weight), medical history, and concomitant medications will be collected from each participant at study initiation. The investigators will record blood pressure, body temperature, respiratory rate, and pulse rate during visits 0 and 1. All adverse events will be comprehensively documented in case report forms, and the association between adverse events and interventions will be categorized as unrelated, possibly related, or related; severity will be classified as mild, moderate, or severe. In the event of serious adverse events, including death, life-threatening incidents, or events requiring hospitalization, the investigator will suspend part or all of the clinical trial and notify the coinvestigators and the IRB within 24 hours. Furthermore, as the trial may primarily involve minors, researchers will implement proactive measures to predict and mitigate the risks associated with potential noncompliance among minor participants.

#### Drop Out Criteria

Participants will be withdrawn from the study if neither fNIRS nor EEG can be performed, if a violation of the inclusion or exclusion criteria is discovered during the study, or if continuing the study becomes difficult owing to the development of adverse or serious adverse events.

Additional criteria for withdrawal include the discovery of a systemic disease not identified before study initiation, any significant violation of the study protocol, or withdrawal of consent by the participant. Furthermore, participants will be excluded if they take medications during the study period that could potentially affect the results, or in any other case in which the investigator determines that proceeding with the study is inappropriate.

#### Statistical Analysis

Statistical analyses will include data from participants who have completed either fNIRS or EEG scans, along with clinical assessments of ASD. Categorical data will be presented as frequencies or percentages and analyzed using chi-square or Fisher exact tests. Numerical data will be reported as means and SDs, with comparisons conducted via independent *t* tests or Wilcoxon rank-sum tests, depending on normality.

To identify differences in frontal network patterns by disease status, 2-sample *t* tests will be conducted to compare network metrics (local efficiency and clustering coefficient) and relative band power between ASD and TD groups.

To examine the interaction effect of acupuncture within this stratified 2×2 design, a 2-way analysis of covariance will be conducted, with the postintervention values of each metric as the dependent variable and the corresponding preintervention values as a covariate. Disease group (ASD vs TD) and intervention type (verum vs sham) will be included as between-participants factors. Within this analysis, the group × intervention interaction will be the primary term of interest, indicating whether verum acupuncture modulates brain networks differently from sham depending on disease status. In addition, as an exploratory analysis, within-group paired *t* tests will be performed to examine pre-to-post changes in each disease group (ASD and TD), separately for the verum and sham conditions.

To investigate the neural substrates of ASD, partial correlation analyses will be conducted between connectivity and network metrics, and clinical scores (K-CARS-2, SRS-2, and REVT), with age and sex included as covariates.

For all brain connectivity analyses (*t* test and partial correlation), statistical significance will be assessed using both an uncorrected threshold of *P*<.001 and a false discovery rate–corrected threshold of *P*<.05 [45]. In contrast, network metrics and relative band power, both expressed as scalar values, will be evaluated at a conventional threshold of *P*<.05.

Statistical analysis will primarily be conducted according to the intention-to-treat principle, including all randomized participants who completed the baseline assessment. To address missing data, we will use multiple imputation techniques for clinical outcomes, provided the data are missing at random. For neuroimaging data, participants exhibiting excessive motion artifacts that remain uncorrectable after preprocessing will be excluded from the final analyses. Additionally, a per-protocol analysis will be performed as a sensitivity check to confirm the consistency of the primary findings.

#### Data Management

The investigators will collect and record medical information on each participant’s case report form. As this study involves minors, informed consent will be obtained from both participants and their legal representatives. In addition, all study data, including patient confirmations, original signed informed consent forms, and detailed primary records, will be retained by the study institution for 3 years following the conclusion of the trial.

#### Quality Control

Important protocol modifications must be reported to the IRB. The initial approval period for the trial was 1 year, requiring regular reports and reapproval for subsequent periods. Prior to the study, all investigators involved in the intervention received comprehensive training in the clinical process. Two independent research assistants will crosscheck the data and case report forms to prevent errors. Currently, there are no plans for early termination of the study before December 2026. Once the target number of participants is reached, additional recruitment may proceed depending on the status of research funding.

### Ethical Considerations

This study will be conducted in accordance with the principles of the Declaration of Helsinki. This trial was approved by the IRB of Sangji University (1040782‐250213-HR-12-148). The protocol was registered with the Clinical Research Information Service of the Republic of Korea (Number: KCT0010437), one of the World Health Organization International Clinical Trials Registry Platform Primary Registries. Written informed consent will be obtained from each participant or their legal representative. To ensure privacy, all data will be anonymized, with identification information recorded using an identification code, and kept confidential in a securely locked location. Participants in this study will receive a small compensation fee of approximately KRW 60,000 (KRW 1=US $0.00075) for their participation.

### Dissemination Plan

The findings of this study will be disseminated through publication in a peer-reviewed journal and presentation at academic conferences in the fields of complementary medicine and neuroimaging. Results will also be shared with clinicians and researchers involved in ASD care, and will be communicated to participating families in an accessible format. The results will be reported regardless of the direction of the findings, including null or negative results, to ensure transparency and reduce publication bias.

## Results

Recruitment and enrollment for the study began in April 2025. To date, 44 participants have been enrolled, comprising 23 individuals in the ASD group and 21 in the TD group. Within the ASD group, 12 (52%) participants were identified as having high-functioning autism, and one was diagnosed with Asperger syndrome. All but 1 of the participants in the ASD group were male. Participant recruitment is currently ongoing. For the TD group, participants were enrolled by matching age and sex to those of the participants with ASD. The protocol currently used in this study is V.1.3 (2025.04.21). This study will continue until December 2026, after which the results will be reported in accordance with the CONSORT (Consolidated Standards of Reporting Trials) checklist.

## Discussion

### Principal Findings

This protocol describes a randomized, sham-controlled trial investigating disease-related alterations and the acute neural mechanisms underlying the effects of acupuncture stimulation on frontal brain networks in patients with ASD and TD, using fNIRS and EEG. We anticipate 3 principal findings.

First, as the primary comparison, we expect that participants with ASD will exhibit altered resting-state frontal network parameters and relative alpha and gamma power compared with TD individuals, consistent with the frontal dysfunction repeatedly reported in ASD.

Second, we anticipate that verum acupuncture will modulate these frontal networks differently from sham acupuncture and that the pattern of modulation will differ between the ASD and TD groups.

Third, we expect that the neurophysiological markers will be associated with clinical symptom severity, as assessed by the K-CARS 2, SRS 2, and REVT, thereby identifying candidate neural substrates of ASD.

### Comparison With Prior Work

Our approach builds on and extends prior neuroimaging work in ASD. Previous fMRI, fNIRS, and EEG studies have consistently reported atypical frontal function in ASD, including altered anterior-posterior connectivity, reduced or dispersed prefrontal connectivity, and altered default-mode network organization [46-48]. At the spectral level, meta-analytic evidence indicates that reduced relative α power and increased γ power are among the most robust resting-state EEG findings in ASD, which motivated our focus on these 2 bands [30]. By combining fNIRS and EEG, our protocol leverages the complementary strengths of hemodynamic and electrophysiological measures to characterize frontal network function.

Unlike most prior work, our study focuses specifically on the frontal region and examines the acute neural response to acupuncture stimulation. Although acupuncture has been clinically applied to individuals with ASD, its immediate effects on brain networks and whether these effects differ by disease status remain largely unexplored.

### Innovations and Limitations

To our knowledge, this study represents the first attempt to use 2 modalities, fNIRS and EEG, to investigate the neural mechanisms of acupuncture stimulation in individuals with ASD compared with TD controls. Within the framework of convergent research, integrating these modalities may capture complex, multidimensional neural responses that a single imaging technique cannot fully reveal, contributing to a mechanistic understanding that informs future evidence-based approaches in integrative medicine.

Despite these innovations, several limitations must be acknowledged. First, because this study examines only the acute neural response to a single acupuncture session, it cannot address the long-term clinical effects or mechanisms of acupuncture treatment in ASD; the acute response may not directly reflect long-term therapeutic outcomes or neural mechanisms, and this association could be examined in future longitudinal studies. Second, expanding the sample size in future investigations will be essential to enhance the statistical power and generalizability of the data.

### Future Directions

Several directions may extend this work. First, recruiting a larger number of participants would increase the statistical power of the study. Second, simultaneous EEG-fNIRS acquisition with whole-brain coverage would enable a more direct investigation of neurovascular coupling and multimodal integration. Third, longitudinal designs incorporating repeated acupuncture sessions would allow exploration of the clinical effects of acupuncture treatment and their underlying mechanisms. Finally, stratification or normative-modeling approaches could address the heterogeneity of ASD and inform the development of individualized acupuncture protocols tailored to a participant’s brain network profile.

### Conclusions

This protocol represents the first randomized, sham-controlled investigation of the acute effects of acupuncture on frontal brain networks in children with ASD, using both fNIRS and EEG. By characterizing disease-related differences in frontal network organization and examining how these networks respond to acupuncture stimulation, this study aims to provide mechanistic insight into acupuncture in ASD and to identify candidate neural markers of the disorder. Elucidating the acute neural response to acupuncture will provide a pivotal foundation for further exploration of its long-term mechanisms in this population. These findings may contribute to future biomarker-driven and individualized approaches to acupuncture intervention in children with ASD.

## Supplementary material

10.2196/97910 Checklist 1 SPIRIT checklist.

## Abbreviations

ASD: autism spectrum disorder
CONSORT: Consolidated Standards of Reporting Trials
EEG: electroencephalogram
fMRI: functional magnetic resonance imaging
fNIRS: functional near-infrared spectroscopy
IRB: institutional review board
K-CARS 2: Korean Childhood Autism Rating Scale 2
MRI: magnetic resonance imaging
REVT: Receptive and Expressive Vocabulary Test
ROI: region of interest
SPIRIT: Standard Protocol Items: Recommendations for Interventional Trials
SRS 2: Social Responsiveness Scale 2
TD: typically developing

## References

[1] Hodges H, Fealko C, Soares N (2020). Autism spectrum disorder: definition, epidemiology, causes, and clinical evaluation. Transl Pediatr.

[2] Lord C, Elsabbagh M, Baird G, Veenstra-Vanderweele J (2018). Autism spectrum disorder. Lancet.

[3] Warrier V, Greenberg DM, Weir E (2020). Elevated rates of autism, other neurodevelopmental and psychiatric diagnoses, and autistic traits in transgender and gender-diverse individuals. Nat Commun.

[4] Baxter AJ, Brugha TS, Erskine HE, Scheurer RW, Vos T, Scott JG (2015). The epidemiology and global burden of autism spectrum disorders. Psychol Med.

[5] Lin D, Shi Z, Hao Z (2025). Global burden, inequality, and frontier gaps of autism spectrum disorder disability in adolescents and young adults, 1990-2021: a systematic analysis of the GBD 2021 study. Front Public Health.

[6] Aishworiya R, Valica T, Hagerman R, Restrepo B (2022). An update on psychopharmacological treatment of autism spectrum disorder. Neurotherapeutics.

[7] Fieiras C, Chen MH, Escobar Liquitay CM (2023). Risperidone and aripiprazole for autism spectrum disorder in children: an overview of systematic reviews. BMJ Evid Based Med.

[8] Howlin P, Magiati I, Charman T (2009). Systematic review of early intensive behavioral interventions for children with autism. Am J Intellect Dev Disabil.

[9] Virués-Ortega J (2010). Applied behavior analytic intervention for autism in early childhood: meta-analysis, meta-regression and dose-response meta-analysis of multiple outcomes. Clin Psychol Rev.

[10] Yu Q, Li E, Li L, Liang W (2020). Efficacy of interventions based on applied behavior analysis for autism spectrum disorder: a meta-analysis. Psychiatry Investig.

[11] Virues-Ortega J, Julio FM, Pastor-Barriuso R (2013). The TEACCH program for children and adults with autism: a meta-analysis of intervention studies. Clin Psychol Rev.

[12] Wong HHL, Smith RG (2006). Patterns of complementary and alternative medical therapy use in children diagnosed with autism spectrum disorders. J Autism Dev Disord.

[13] Owen-Smith AA, Bent S, Lynch FL (2015). Prevalence and predictors of complementary and alternative medicine use in a large insured sample of children with autism spectrum disorders. Res Autism Spectr Disord.

[14] Lee B, Lee J, Cheon JH, Sung HK, Cho SH, Chang GT (2018). The efficacy and safety of acupuncture for the treatment of children with autism spectrum disorder: a systematic review and meta-analysis. Evid Based Complement Alternat Med.

[15] Chen D, Yang X, Jiao D (2025). Electroacupuncture ameliorates autism spectrum disorder via modulating the gut-brain axis depending on the integrity of vagus nerve. Transl Psychiatry.

[16] Chen S, Huang L, Liu G (2023). Acupuncture ameliorated behavioral abnormalities in the autism rat model via pathways for hippocampal serotonin. Neuropsychiatr Dis Treat.

[17] Li J, Liu L, Sun S, Liu C, Yin P (2026). BDNF and miRNAs in acupuncture-regulated neuroplasticity: emerging mechanisms and therapeutic strategies. Medicine (Baltimore).

[18] Yau CH, Ip CL, Chau YY (2018). The therapeutic effect of scalp acupuncture on natal autism and regressive autism. Chin Med.

[19] Wong VCN, Chen WX (2010). Randomized controlled trial of electro-acupuncture for autism spectrum disorder. Altern Med Rev.

[20] Ecker C, Bookheimer SY, Murphy DGM (2015). Neuroimaging in autism spectrum disorder: brain structure and function across the lifespan. Lancet Neurol.

[21] Patil M, Iftikhar N, Ganti L (2024). Neuroimaging insights into autism spectrum disorder: structural and functional brain. Health Psychol Res.

[22] Mokienko OA (2025). The potential of near-infrared spectroscopy as a therapeutic tool following a stroke (review). Sovrem Tekhnologii Med.

[23] Boas DA, Elwell CE, Ferrari M, Taga G (2014). Twenty years of functional near-infrared spectroscopy: introduction for the special issue. Neuroimage.

[24] Hernandez-Pavon JC, Veniero D, Bergmann TO (2023). TMS combined with EEG: recommendations and open issues for data collection and analysis. Brain Stimul.

[25] Zhang F, Roeyers H (2019). Exploring brain functions in autism spectrum disorder: a systematic review on functional near-infrared spectroscopy (fNIRS) studies. Int J Psychophysiol.

[26] Zhu H, Fan Y, Guo H, Huang D, He S (2014). Reduced interhemispheric functional connectivity of children with autism spectrum disorder: evidence from functional near infrared spectroscopy studies. Biomed Opt Express.

[27] Sun W, Wu X, Zhang T, Lin F, Sun H, Li J (2021). Narrowband resting-state fNIRS functional connectivity in autism spectrum disorder. Front Hum Neurosci.

[28] Garcés P, Baumeister S, Mason L (2022). Resting state EEG power spectrum and functional connectivity in autism: a cross-sectional analysis. Mol Autism.

[29] Kakuszi B, Szuromi B, Tóth M, Bitter I, Czobor P (2024). Alterations in resting-state gamma-activity in adults with autism spectrum disorder: a high-density EEG study. Psychiatry Res.

[30] Neo WS, Foti D, Keehn B, Kelleher B (2023). Resting-state EEG power differences in autism spectrum disorder: a systematic review and meta-analysis. Transl Psychiatry.

[31] Chan AW, Tetzlaff JM, Gøtzsche PC (2013). SPIRIT 2013 explanation and elaboration: guidance for protocols of clinical trials. BMJ.

[32] Kwon HJ, Yoo HJ, Kim JH (2017). Re-adjusting the cut-off score of the Korean version of the Childhood Autism Rating Scale for high-functioning individuals with autism spectrum disorder. Psychiatry Clin Neurosci.

[33] Chun J, Bong G, Han JH, Oh M, Yoo HJ (2021). Validation of Social Responsiveness Scale for Korean preschool children with autism. Psychiatry Investig.

[34] Carty A, Green R, Goodman CV (2025). Performance of the Social Responsiveness Scale-2 for the assessment of autistic behaviors in a sample of Canadian preschool-aged children. J Autism Dev Disord.

[35] Kwon S, Sung IY, Ko EJ, Kim HS (2019). Effects of therapeutic horseback riding on cognition and language in children with autism spectrum disorder or intellectual disability: a preliminary study. Ann Rehabil Med.

[36] Shin JH, Kang MJ, Lee SA (2024). Wearable functional near-infrared spectroscopy for measuring dissociable activation dynamics of prefrontal cortex subregions during working memory. Hum Brain Mapp.

[37] Pu Z, Huang H, Li M (2024). An exploration of distinguishing subjective cognitive decline and mild cognitive impairment based on resting-state prefrontal functional connectivity assessed by functional near-infrared spectroscopy. Front Aging Neurosci.

[38] Delorme A, Mullen T, Kothe C (2011). EEGLAB, SIFT, NFT, BCILAB, and ERICA: new tools for advanced EEG processing. Comput Intell Neurosci.

[39] Li X, Li JC, Lu QQ, Zhang F, Zhang SQ (2023). Research status and prospects of acupuncture for autism spectrum disorders. Front Psychiatry.

[40] Kim TH, Lee MS, Lee H (2022). Sham acupuncture is not just a placebo. J Acupunct Meridian Stud.

[41] Wang Z, Zhang J, Zhang Z (2024). Effects of acupuncture for Bell’s palsy patients in the acute phase and its impact on facial nerve edema: a study protocol for a randomized, controlled trial. Front Neurol.

[42] Yu C, Zhang R, Shen B (2023). Effects of sham acupuncture for chronic musculoskeletal pain syndrome: a systematic review and network meta-analysis of randomized controlled trials. Medicine (Baltimore).

[43] Xu S, Yu L, Luo X (2020). Manual acupuncture versus sham acupuncture and usual care for prophylaxis of episodic migraine without aura: multicentre, randomised clinical trial. BMJ.

[44] Lee H, Bang H, Kim Y (2011). Non-penetrating sham needle, is it an adequate sham control in acupuncture research?. Complement Ther Med.

[45] Benjamini Y, Hochberg Y (1995). Controlling the false discovery rate: a practical and powerful approach to multiple testing. J R Stat Soc Series B Stat Methodol.

[46] Just MA, Keller TA, Malave VL, Kana RK, Varma S (2012). Autism as a neural systems disorder: a theory of frontal-posterior underconnectivity. Neurosci Biobehav Rev.

[47] Lynch CJ, Uddin LQ, Supekar K, Khouzam A, Phillips J, Menon V (2013). Default mode network in childhood autism: posteromedial cortex heterogeneity and relationship with social deficits. Biol Psychiatry.

[48] Yeung MK, Lee TL, Chan AS (2019). Frontal lobe dysfunction underlies the differential word retrieval impairment in adolescents with high-functioning autism. Autism Res.

